# Fish diversity of a spring field in Hopong Town, Taunggyi District, Shan State, Myanmar (the Salween River Basin), with genetic comparisons to some “species endemic to Inle Lake”

**DOI:** 10.3897/BDJ.10.e80101

**Published:** 2022-04-05

**Authors:** Yuichi Kano, Yusuke Fuke, Prachya Musikasinthorn, Akihisa Iwata, Tin Mya Soe, Sein Tun, LKC Yun, Seint Seint Win, Shoko Matsui, Ryoichi Tabata, Katsutoshi Watanabe

**Affiliations:** 1 Kyushu Open University, Fukuoka, Japan Kyushu Open University Fukuoka Japan; 2 Kyushu University, Fukuoka, Japan Kyushu University Fukuoka Japan; 3 Kyoto University, Kyoto, Japan Kyoto University Kyoto Japan; 4 Kasetsart University, Bangkok, Thailand Kasetsart University Bangkok Thailand; 5 Inlay Lake Wildlife Sanctuary, Nyaung Shwe, Myanmar Inlay Lake Wildlife Sanctuary Nyaung Shwe Myanmar; 6 Natma Taung National Park, Kanpetlet, Myanmar Natma Taung National Park Kanpetlet Myanmar; 7 Hkakaborazi National Park, Putao, Myanmar Hkakaborazi National Park Putao Myanmar; 8 Taunggyi University, Taunggyi, Myanmar Taunggyi University Taunggyi Myanmar; 9 Kyaing Tong University, Keng Tung, Myanmar Kyaing Tong University Keng Tung Myanmar; 10 Osaka Museum of Natural History, Osaka, Japan Osaka Museum of Natural History Osaka Japan; 11 Lake Biwa Museum, Kusatsu, Japan Lake Biwa Museum Kusatsu Japan

**Keywords:** Burma, *Inlecyprisauropurpureus*, *Microrasborarubescens*, mitochondrial DNA (mtDNA), *Physoschisturabrunneana*, *Sawbwaresplendens*

## Abstract

Hopong, a small town in the Salween (Thanlwin) River Basin, Myanmar, is located 35 km northeast of Inle Lake, a famous ancient lake with numerous endemic fish species. We surveyed the fish fauna of a spring pond in Hopong in 2016, 2019 and 2020 and identified 25 species. Of these, seven, including *Inlecyprisauropurpureus* and *Sawbwaresplendens*, had been considered endemic to Inle Lake and at least three species were genetically unique. Eight were suspected or definite introduced species, including *Oreochromisniloticus* and *Gambusiaaffinis*. We were unable to identify a nemacheilid species of the genus *Petruichthys*, which would need a taxonomic examination. The Hopong area is being developed rapidly and, hence, it is crucial to conserve its native fish species and the freshwater ecosystems.

## Introduction

**Figure 1.**
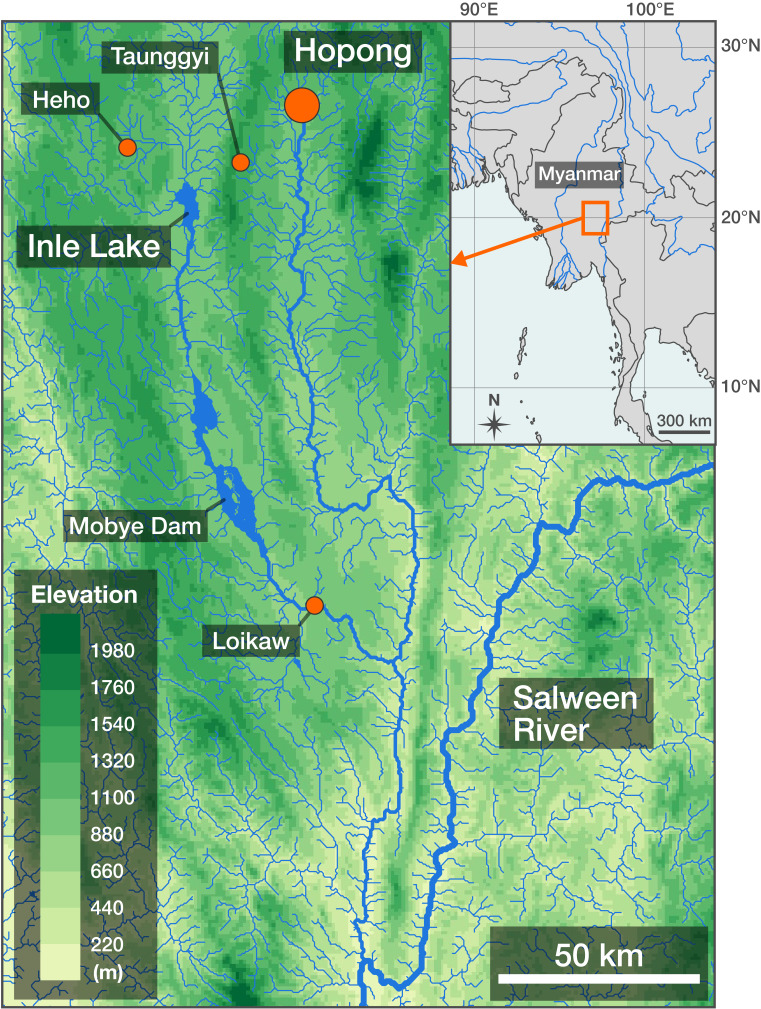
A map of Hopong and the surrounding region.

Hopong is a small town in the Salween (Thanlwin) River Basin, situated 15 km east of Taunggyi, the capital of Shan State, Myanmar (Fig. 1). The town has several natural wetlands and springs. Danio (Celestichthys) margaritatus, a minute cyprinid fish, is a symbolic fish species from this area (Roberts 2007, Conway et al. 2008). However, the fields of Hopong have been developed rapidly with extensive road construction and urbanisation (Roberts 2007).

Hopong is about 35 km northeast of Inle Lake, a famous ancient lake with many endemic fish species (Annandale 1918, Kano et al. 2016, Win 2018). The Hopong area and Inle Lake are both in the Salween River Basin, so are close together geographically; however, they belong to different drainages separated by highlands and are ca. 400 km apart by river (Fig. 1). The endemism of fishes in Inle Lake has not been validated, because studies of the fish fauna of the middle–upper Salween Basin are insufficient. Thus, the ichthyofauna of the Hopong area is of biogeographical interest, especially in terms of comparison with that of Inle Lake, including the validation of species endemic to Inle Lake.

Generally, studies of freshwater fish biodiversity in Myanmar are insufficient; most are fragmentary reports on fauna of specific regions or new species. Annandale (1918) and Hora and Mukerji (1934) reported the fish fauna of Inle Lake and Shan State, respectively. Musikasinthorn (1998a) first reported *Channapanaw* from the Irrawaddy and Sittang River Basins, Myanmar. Britz (2003) described a new species of miniature fish, *Danionellamirifica*, from northern Myanmar. Roberts (2012) reported a new species of arowana, *Scleropagesinscriptus*, from the Malay Peninsula, Myanmar. Recently, Vidthayanon et al. (2005) provided a rough list of inland fish species of Myanmar. Data on the freshwater fish biodiversity of Myanmar are generally limited and more information is required.

Here, we report the results of a freshwater fish inventory for a spring field in Hopong conducted in 2016, 2019 and 2020. The list contains 25 species, including seven “species endemic to Inle Lake”. We provide DNA barcoding (mitochondrial COI sequences) data for the species, which clarify their genetic uniqueness and should promote biogeographical research in this area.

## Methods

**Figure 2.**
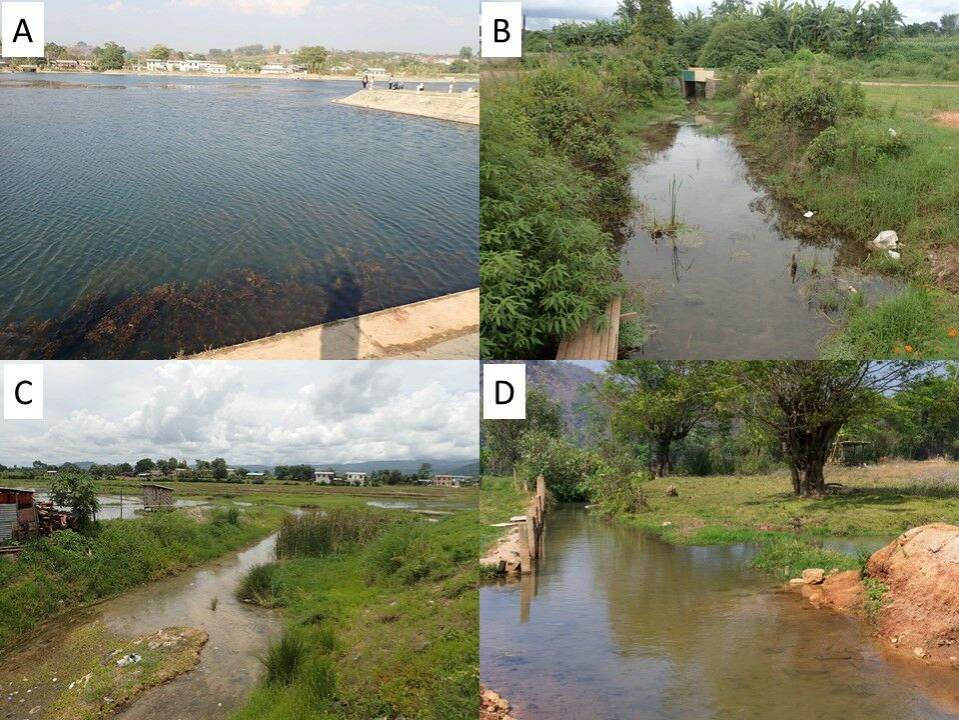
Photos of the spring field in Hopong. **A** A small dam holding spring water, inhabited by *Sawbwaresplendens* and *Oreochromisniloticus.* **B** A shallow dam backwater, inhabited by *Daniomargaritatus* and *D.erythromicron.* **C** A stream flowing from the dam, inhabited by *Devariosondhii* and *Inlecyprisauropurpureus.* **D** A ditch and shallow wetland, inhabited by *Physoschisturarivulicola* and *Channaharcourtbutleri*.

Sampling was conducted nine times in Hopong from March 2016 to March 2020 (Fig. 2). Fish were collected with hand-nets, throwing nets and fish traps. Except for several individuals that were only photographed or recorded on-site and released, the collected specimens were photographed fresh (Kano and Nakajima 2014), fixed in 10% formalin and then transferred to 70% ethanol. The specimens and their tissue samples were catalogued and deposited at the Research Laboratory of Ichthyology, Department of Fishery Biology, Faculty of Fisheries, Kasetsart University, Bangkok, Thailand (RLIKU) and Kyoto University, Kyoto, Japan (tissue samples). All specimens were assigned IDs associated with the records of location (latitude, longitude and region name), collection date, DNA sequence accession numbers etc. The data were registered in the integrated Monsoon Asia “ffish.asia” online database of freshwater organism biodiversity (Watanabe et al. 2009, Kano et al. 2013) and can be retrieved at https://ffish.asia/Hopong2020.

To obtain DNA barcoding data (partial mitochondrial COI sequences) for the species collected from Hopong (102 specimens from 19 species including all native species), total DNA was extracted using a Genomic DNA Purification kit (Promega) or Monarch Genomic DNA Purification Kit (New England Biolabs). The COI gene was amplified by PCR using the primer pair FishF1 (5′-TCA ACC AAC CAC AAA GAC ATT GGC AC-3′) and FishR1 (5′-TAG ACT TCT GGG TGG CCA AAG AAT CA-3′) (Ward et al. 2005). The amplification consisted of an initial denaturation step (94°C, 2 min); 30 cycles of 94°C for 30 s, 56–60°C for 30 s and 72°C for 60 s; and a final extension (72°C, 7 min). The PCR products were purified with Illustra ExoStar (GE Healthcare) and sequenced on an ABI 3130xl Genetic Analyzer (Applied Biosystems) using an amplification primer and the BigDye Terminator Cycle sequencing FS Ready Reaction kit ver. 3.1 (Applied Biosystems). The DNA sequences (640 bp) were deposited at DDBJ/EMBL/GenBank (accession numbers: LC190268–LC190330, LC190383, LC190395–LC190405, LC645163–LC645189).

The obtained sequences were aligned using MAFFT (Katoh and Standley 2013) at Unipro UGENE (Okonechnikov et al. 2012). Sequence data for the same or related species from Inle Lake and the surrounding rivers, as reported by Kano et al. (2016), were included in the analysis for comparison with the Hopong populations. To visualise the relationships amongst populations, haplotype networks, based on the TCS algorithm (statistical parsimony; Clement et al. 2000), were constructed for five genera endemic to this region—*Inlecypris*, *Microrasbora*, *Sawbwa*, *Petruichthys* and *Physoschistura*—using POPART (Leigh and Bryant 2015). The mean uncorrected sequence differences amongst different populations were calculated using MEGA 7 (Kumar et al. 2016).

## Results

The survey uncovered 25 species (Table 1). We collected *Daniomargaritatus* in a shallow wetland habitat, as described by Roberts (2007). We also obtained *Devariosondhii*, which was reported by *Hora and Mukerji (1934)* with a specimen obtained in the Hopong area. We also found a likely undescribed species of *Petruichthys*, which we tentatively labelled “*Petruichthys* sp. (Hopong)”. Of the 25 species, seven had been treated as “species endemic to Inle Lake”: *Danioerythromicron*, *Inlecyprisauropurpureus*, *Microrasborarubescens*, *Sawbwaresplendens*, *Petruichthysbrevis*, *Channaharcourtbutleri* and *Mastacembeluscaudiocellatus* (Annandale 1918, Kano et al. 2016). Eight were suspected or definite introduced species: *Cyprinusrubrofuscus*, *Esomusdanrica*, *Parambassislala*, *Oreochromisniloticus*, *Trichogasterlabiosa*, *Oryziasuwai*, *Gambusiaaffinis* and *Poeciliareticulate* (Annandale 1918, Hora and Mukerji 1934, Musikasinthorn 1998b, Kano et al. 2016). Three of these (i.e. *E.danrica*, *T.labiosa*, *O.uwai*) are distributed widely in Southeast Asia; hence the possibility that these species are native cannot be completely ruled out.

**Figure 3.**
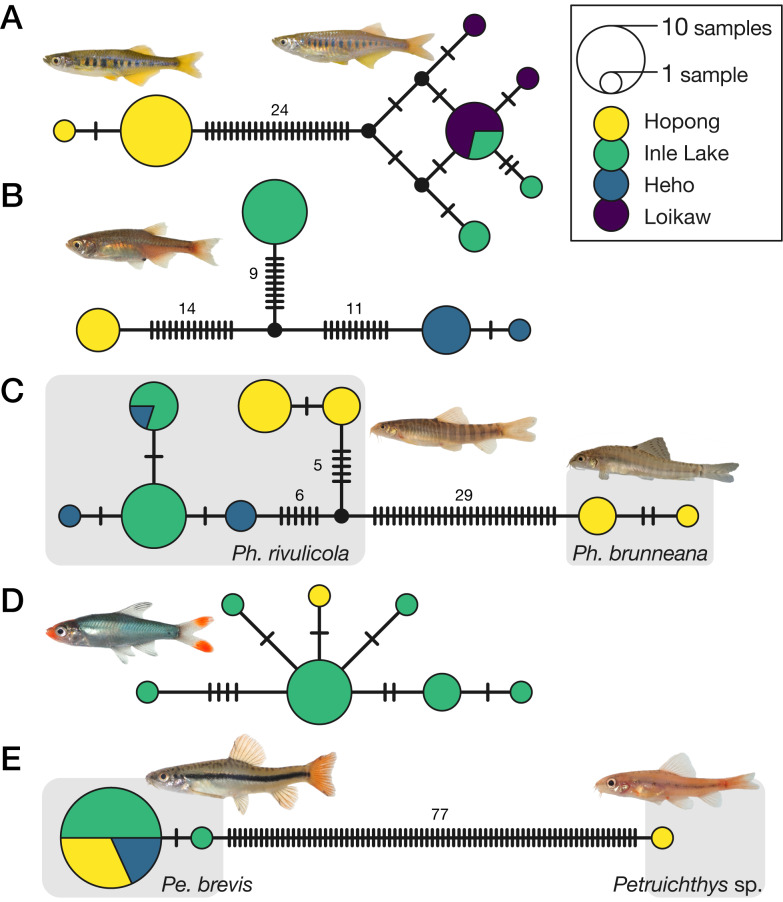
The haplotype networks of five species groups (“species endemic to Inle Lake”) generated with the TCS algorithm, based on COI regions (640 bp). Each bar on a branch corresponds to a single nucleotide substitution. Small dots represent hypothetical haplotypes. **A** *Inlecyprisauropurpureus* obtained from Hopong and two other localities. **B** *Microrasborarubescens* obtained from Hopong and two other localities. The Heho specimens were obtained from a local market and their original locality is unknown. **C** *Physoschisturabrunneana* and *P.rivulicola* obtained from Hopong and two other localities. The Heho specimens were obtained from a local market and their original locality is unknown. **D** *Sawbwaresplendens* obtained from Hopong and Inle Lake. **E** *Petruichthysbrevis* obtained from Hopong and two other localities and *Petruichthys* sp. obtained from Hopong.

Haplotype networks of the populations of three species from the Hopong area (*Inlecyprisauropurpureus* [Fig. 3A], *Microrasborarubescens* [Fig. 3B] and *Physoschisturarivulicola* [Fig. 3C]) showed genetic uniqueness compared to Inle Lake populations, even within species. *Inlecyprisauropurpureus*, *Microrasborarubescens* and *Physoschisturarivulicola* showed clear genetic divergence (4.1%, 3.2% and 2.1%, respectively, in mean uncorrected sequence differences). By contrast, *Sawbwaresplendens* (Fig. 3D) and *Petruichthysbrevis* (Fig. 3E) showed no clear genetic divergence amongst local populations (0.3% and < 0.1%, respectively). *Petruichthys* sp. (Hopong) showed an obviously distinct genetic profile from the sympatric *P.brevis* (12.6% mean uncorrected sequence difference; Fig. 3E).

## Discussion

A total of eleven freshwater fish species have been reported from Hopong in previous papers (Hora and Mukerji 1934, Roberts 2007, Kullander et al. 2017). All of them, except for *Barbushexastichus* (valid as *Neolissochilushexastichus*), are judged to be included in the 25 species reported in this study. Hora and Mukerji (1934) reported *Barbushexastichus* from Hopong as having two pairs of barbels, a light brown body colour and a black round spot at the base of the caudal fin. However, this record should be reconsidered since its main distribution range is in India (Talwar and Jhingran 1991). In fact, *Neolissochilusnigrovittatus*, which is distributed around Inle Lake (Talwar and Jhingran 1991, Kano et al. 2016), also fits the description of the fish by Hora and Mukerji (1934). Further survey and examination are necessary for the *Neolissochilus* species in this area.

Our results indicated that at least seven “species endemic to Inle Lake” (Annandale 1918, Kano et al. 2016, Win 2018) have wider distributions than previously thought, beyond the Inle Lake Basin (Table 1). Artificial introduction from Inle Lake cannot explain their wide distributions because of the genetic divergence of the Hopong populations from the Inle Lake populations, except for *Sawbwaresplendens* (only one DNA sample and details unknown) and *Petruichthysbrevis* (Fig. 3D and E). Therefore, Hopong shares fish fauna with the Inle Lake Basin, but the populations of several fish species in Hopong are genetically unique and require detailed taxonomic examinations.

*Daniomargaritatus* is symbolic of Hopong (Roberts 2007). Conway et al. (2008) reported that *D.margaritatus* is distributed not only in Hopong (the type locality of the species; Roberts 2007), but also in wider areas of Myanmar and Thailand following Clarke (2007) and Hary (2007). However, these sources are not formal scientific reports (website and local report, respectively) and, in fact, we could not access them. Thus, at present, it cannot be determined whether *D.margaritatus* is distributed only in the Hopong area or more widely. If the former is the case, conservation of *D.margaritatus* and its habitat, i.e. shallow ponds/wetlands with plants and clear water from springs (Fig. 2B; Roberts 2007), is very important. *Devariosondhii* might also be endemic to the Hopong area; more information on its distribution is necessary to determine its conservation status.

*Petruichthys* sp. (Hopong) is genetically distinguishable from the sympatric *P.brevis* (Fig. 3E). Morphologically, its body is smaller and the male is redder than that of *P.brevis*. Taxonomic examination of this fish is required.

The Hopong area is being developed rapidly (Roberts 2007). In addition, several invasive alien fish have become established, including *Oreochromisniloticus* and *Gambusiaaffinis*. This strongly suggests that the native freshwater fishes and other native freshwater organisms in Hopong are now severely threatened. As explained above, Hopong has a unique fish fauna that includes endemic species and species shared with Inle Lake, an unique ancient lake in Southeast Asia. Therefore, wetland and biodiversity conservation in Hopong are a high priority.

## Figures and Tables

**Table 1.** Fish species collected in a spring field in Hopong, the Salween Basin, Myanmar

**Order**	**Family**	**Species**	***N* (DNA barcoding)**	**Accession number**
Cypriniformes				
	Cyprinidae	*Cyprinusrubrofuscus* ^*^	1 (0)	—
		*Danioerythromicron* ^†^	33 (12)	LC190280, LC190284–LC190286, LC190314, LC645170–LC645175, LC645189
		*Daniomargaritatus*	21 (9)	LC190268, LC190311–LC190313, LC190315, LC190395–LC190398
		*Devariobrawni*	6 (4)	LC190317, LC190318, LC190320, LC645169
		*Devariosondhii*	21 (20)	LC190281, LC190289–LC190304, LC645177–LC645179
		*Esomusdanrica* ^*^	2 (1)	LC645176
		*Inlecyprisauropurpureus* ^†^	19 (13)	LC190269–LC190279, LC645167, LC645168
		*Microrasborarubescens* ^†^	66 (4)	LC190305–LC190308
		*Pethiastoliczkana*	30 (2)	LC190282, LC190283
		*Sawbwaresplendens* ^†^	2 (1)	LC645184
		Systomussp. cf.rubripinnis	3 (2)	LC190316, LC645185
		*Lepidocephalichthysberdmorei*	9 (3)	LC190324–LC190326
	Nemacheilidae	*Petruichthysbrevis* ^†^	18 (7)	LC190287, LC190288, LC190309, LC190310, LC645165, LC645166, LC645186
		*Petruichthys* sp. (Hopong)	2 (1)	LC645164
		*Physoschisturabrunneana* ^†^	5 (4)	LC190399–LC190402
		*Physoschisturarivulicola*	16 (9)	LC190319, LC190321–LC190323, LC190403–LC190405, LC645181, LC645182
Cyprinodontiformes				
	Poeciliidae	*Gambusiaaffinis* ^*^	1 (1)	LC645188
		*Poeciliareticulata* ^*^	1 (0)	—
Cichliformes				
	Ambassidae	*Parambassislala* ^*^	10 (2)	LC645180, LC645187
	Cichlidae	*Oreochromisniloticus* ^*^	1 (0)	—
Beloniformes				
	Adrianichthyidae	*Oryziasuwai* ^*^	11 (0)	—
Synbranchiformes				
	Synbranchidae	*Monopterusjavanensis*	1 (1)	LC190383
	Mastacembelidae	*Mastacembeluscaudiocellatus* ^†^	2 (1)	LC645163
Anabantiformes				
	Osphronemidae	*Trichogasterlabiosa* ^*^	7 (0)	—
	Channidae	*Channaharcourtbutleri* ^†^	30 (5)	LC190327–LC190330, LC645183
^*^ Putative introduced species; ^†^ Assumed as endemic to Inle Lake				
